# Factors associated with current and severe pain among people living with HIV: results from a statewide sample

**DOI:** 10.1186/s12889-020-09474-y

**Published:** 2020-09-18

**Authors:** Verlin Joseph, Abenaa Jones, Shantrel Canidate, Zachary Mannes, Huiyin Lu, Nichole Ennis, Gladys Ibanez, Charurut Somboonwit, Robert Cook

**Affiliations:** 1 Department of Epidemiology, College of Public Health and Health Professions & College of Medicine, University of Florida, P.O. Box 100231, 2004 Mowry Road, Gainesville, FL 32610 USA; 2 Department of Health, Behavior, and Society, Bloomberg School of Public Health, Johns Hopkins University, 615 N Wolfe St, Baltimore, MD 21205 USA; 3 Department of Clinical and Health Psychology, College of Public Health and Health Professions, University of Florida, P.O. Box 100231, 2004 Mowry Road, Gainesville, FL 32610 USA; 4 Fred Hutchinson Cancer Research Center, University of Washington, 1100 Fairview Ave N, Seattle, WA 98109 USA; 5 Department of Behavioral Sciences and Social Medicine, Center for Translational Behavioral Sciences, College of Medicine, Florida State University, 2010 Levy Ave, Bldg. B, suite 266, Tallahassee, FL 32310 USA; 6 Department of Epidemiology, Florida International University, 11200 S.W. 8th Street, Building AHC-5, Room 478, Miami, Florida 33199 USA; 7 Division of Infectious Disease and International Medicine, Morsani College of Medicine, University of South Florida, 12901 Bruce B Downs Blvd, Tampa, FL 33612 USA

**Keywords:** Marijuana, Pain, PLHIV, Mental health, Substance use

## Abstract

**Background:**

People living with HIV (PLHIV) are more likely to suffer from pain compared to the general public. Pain often clusters with mental health symptoms and substance use. This study sought to evaluate mental health and substance use factors associated with any pain and severe pain intensities among PLHIV.

**Methods:**

Data were derived from HIV+ adults (*N* = 733) recruited from community health centers across Florida who completed questionnaires regarding demographics, chronic pain, HIV clinical outcomes, mental health symptoms, and substance use information. Pain was assessed using the Brief Pain Inventory (BPI) short form. Multivariate logistic regression analysis was utilized to assess the relationship between selected covariates and pain.

**Results:**

Approximately half (45.0%) of participants reported having any current pain while 16.1% reported severe pain. The odds of having any current pain were 2.49 (CI 95% 1.48, 4.18, *p* <  0.01) times greater among PLHIV reporting anxiety and 1.69 (CI 95% 1.11, 2.57, *p* = 0.01) times greater among PLHIV reporting PTSD compared to those without those factors. The odds of having severe pain were 2.03 (CI 95% 1.03, 4.01, *p* = 0.04) times greater among PLHIV reporting anxiety and 2.02 (CI 95% 1.26, 3.24, *p* <  0.01) times greater among female participants compared to PLHIV without those factors respectively. Factors including depression, alcohol consumption, and marijuana use were not statistically associated with any current pain nor with severe pain.

**Conclusion:**

The relationship between pain and mental health is complex. Thus, future research is needed to determine if pain treatments may reduce mental health symptoms or if treatments can be targeted to address both issues simultaneously.

## Background

Advancements in antiretroviral therapy (ART) have increased the life expectancy among people living with HIV/AIDS (PLHIV) and have transformed the once fatal disease into a serious chronic illness [1]. As a result, researchers have shifted their focus to developing a greater understanding of the quality of life among PLHIV within the psychosocial context of chronic pain [2–4]. PLHIV are more likely to suffer from pain associated with musculoskeletal disorders, neuropathic pain, and headache disorders than the general population [5, 6]. Additionally, PLHIV reporting severe pain are more likely to report missed clinic visits compared to PLHIV without pain [7]. As such, identifying factors associated with pain may serve as a crucial first step in improving the quality of life and clinical outcomes among PLHIV.

In addition to chronic pain, PLHIV have also reported mental health and harmful substance use issues [6, 8]. Generally, PLHIV experiencing mental health problems such as anxiety and depression are also more likely to report increases in pain [9, 10]. Traditionally, clinicians have often prescribed opioids to treat pain for PLHIV [11]; however, PLHIV are more likely to report high levels of pain even when using prescription opioids, which have become the leading sources of prescription drug abuse [12–15]. PLHIV reporting substance use and depression are more likely to report a lower quality of life compared to non-users [16]. However, research examining the intersection of substance abuse, mental health, and pain remains limited [6, 10, 17]. Thus, continued research is needed to understand the associations between clustering mental health and substance use factors and pain severity.

Generally, studies examining clustering mental health and substance use factors do not include pain severity [18–20]. Therefore, further research is needed to determine which specific patterns of substance use and mental health predictors are associated with pain. Understanding the intersection of mental health and substance use in the context of pain is vital for developing interventions aligned with the White House’s National HIV/AIDS Strategy of improving health outcomes for PLHIV [21]. Further, identifying factors associated with pain may inform strategies that seek to improve quality of life and improve clinical outcomes among PLHIV [22, 23]. One shared limitation of previous studies targeting pain and substance use among PLHIV was the inability to control for the peak-end phenomenon. The peak-end phenomenon occurs when participants recall the worst pain intensity scores when queried about average pain intensity ratings [24]. As such, it is crucial to include worst pain intensity ratings when studying correlates of pain. Thus, we specifically sought to examine: 1) the prevalence of any current pain and severe pain in a statewide sample of PLHIV; 2) mental health conditions and substance use factors associated with any current pain and with severe pain in PLHIV.

## Methods

### Study design

The Florida Cohort Study is an ongoing statewide prospective longitudinal study that seeks to identify factors that influence health outcomes in PLHIV. This study is an analysis of cross sectional data collected among PLHIV living in Florida. The protocol was approved by the Institutional Review Boards (IRBs) at the University of Florida and the Florida Department of Health (DoH). All participants provided written informed consent as well as signed the Health Insurance Portability and Accountability Act (HIPPA) authorization form for use of their protected medical information.

Eligible participants were adults 18 years or older with a diagnosis of HIV. Participants were recruited from multiple cities in Florida (Gainesville, Ft. Lauderdale, Lake City, Miami, Orlando, Sanford, Tampa, and Wildwood) from 2014 to 2017. The study team’s recruitment efforts entailed posting brochures at community clinics and county health departments, having clinic/facility staff members reach out to potential participants, and consulting patient registries.

### Measures

Participants completed study questionnaires during study enrollment. The questionnaire was developed for this study and has been made available online [25]. Data on demographics, HIV clinical outcomes, mental health, and the use of substances including alcohol, illicit drugs, and marijuana were obtained by self-report. Demographic variables included age, gender at birth (i.e., male or female), race/ethnicity (non-Hispanic Black, Hispanic, and non-Hispanic White), and employment status (employed or unemployed).

The questionnaire inquired about current and lifetime substance use of marijuana, alcohol, injection drug use (IDU), and crack/cocaine. Individuals who indicated having used marijuana during the past 3 months were categorized as current users while individuals abstaining from marijuana use were categorized as nonusers. Participants indicating any IDU or crack/cocaine use during the past year or during any period of time were categorized as lifetime users. Hazardous drinking was defined in concordance with the National Institute on Alcohol Abuse and Alcoholism (NIAAA) definition as consuming ≥14 drinks per week or ≥ 5 drinks per occasion at least monthly for men and ≥ 7 drinks per week or ≥ 4 drinks per occasion at least monthly for women [26].

Based on their self-reported reasons for use, participants who used marijuana were divided into two groups any recreational use and medicinal use only. In this study, recreational use was defined as using marijuana for the purpose of getting high or stoned, increasing libido/improving sexual performance, or fitting into social situations, while medicinal marijuana use was defined as using marijuana for the purpose of improving appetite/gaining weight, inducing sleep, relieving nausea/vomiting, relieving pain, or relieving anxiety/depression/stress. Participants reporting marijuana use for both recreational and medicinal purposes were classified as recreational users. This distinction was made due to prior research suggesting a variety of motives for marijuana use depending on recreational or medicinal use) [27].

This study employed the Brief Pain Inventory short form (BPI-SF) [28] to assess the severity of the participants’ pain and to examine the impact of the experienced pain on each participant’s daily functioning. Using a scale of 1–10, participants were asked to rate their level of pain intensity, other than common pains, during the previous 24 h, identifying the pain level at its worst, least, average, and current. In concordance with the BPI user guide, the average of the four pain level intensity scores reported by the participant provided a composite pain score [29]. PLHIV that did not report any pain during the previous 24 h was categorized as having no current pain. Participants with an average score of ≥1 were categorized as having any current pain [30]. In order to account for the peak-end phenomenon, we utilized a cut-off score for worst pain of ≥6 in the secondary analysis of severe pain [24].

The Generalized Anxiety Disorder Scale (GAD-7) was used to assess participants’ levels of anxiety. The GAD-7 is composed of seven items that screen for the presence of an anxiety disorder. Based on their responses, participants were dichotomized as having no symptoms of anxiety (score 0–9) or current anxiety symptoms (scores ≥10) [31].

Participants also completed the Patient Health Questionnaire (PHQ-8), consisting of 8 items to assess the frequency of depressive symptoms among the participants. Scores can range from 0 to 24, and participants were categorized as having no current depression symptoms (score 1–9), or current depressive symptoms (score ≥ 10). The PHQ-8 has demonstrated high reliability and is a well-validated measure for assessing depressive symptoms among PLHIV [32].

The Primary Care Post Traumatic Stress Disorder Screener (PC-PTSD), which contains 4 items, is a self-reported questionnaire designed to assess symptoms of PTSD. Individuals indicating one or more symptom(s) were coded as positive for PTSD symptoms [33].

### Statistical analyses

Data were analyzed using SAS™ (Statistical Analysis Software) version 9.4 (SAS Institue Inc., Cary, NC). First, we assessed the bivariate relationships of mental health conditions, substance use, and sociodemographic factors associated with pain. For the purposes of this analysis, age was dichotomized 18–49 and ≥ 50 years to improve interpretation of the results. We utilized simple logistic regression modeling to report the unadjusted odds ratios and *p*-values of the associations between these factors and pain scores. Variables that were statistically significant (*p* < .05) in the bivariate analysis were included in the multivariable model. We then used a multivariate logistic regression to distinguish the relationship between mental health, substance use, sociodemographic factors, and any pain. In this multivariate logistic regression model, no pain was assigned as the referenced pain level. We then used a multivariate logistic regression to distinguish the relationship between mental health, substance use, and sociodemographic factors associated with severe pain. In this multivariate logistic regression model, the reference category was pain scored between 0 and 6. All data analyses were conducted using SAS version 9.4.

## Results

The goal of this analysis was to report the prevalence of pain and identify correlates of pain among PLHIV. A total of 733 PLHIV enrolled in the Florida Cohort Study completed the study assessments (Table 1). Roughly half (45.0%) of our sample reported pain in the previous 24 h. Additionally, 118 (16.1%) participants reported severe pain in the previous 24 h. Overall, 426 (58.1%) participants were aged between 18 and 49, and 307 (41.9%) were in the age group of 50 years and older. Furthermore, 500 (68.2%) of participants were men and 233 (31.8%) were women. Among the participants who completed the demographic assessment, 403 (57.3%) were black and roughly 73.8% were unemployed.

**Table 1:** Demographic Mental Health and Substance Use Characteristics of Any Current Pain Among PLHIV *N* = 733

Characteristics	No Pain *N* = 403 (56.0%)	Any Pain *N* = 330 (45.0%)	Unadjusted Odds Ratio	*P* Value
Age in Years				**< 0.01**
18–49 (*n* = 426)	255 (59.9)	171 (40.1)	ref	
≥ 50 (*n* = 307)	148 (48.2)	159 (51.8)	**1.60 (1.19, 2.15)**	
Sex				0.11
Male (*n* = 500)	285 (57.0)	215 (43.0)	ref	
Female (*n* = 233)	118 (50.6)	115 (49.4)	1.29 (0.95,1.77)	
Race/ethnicity				0.15
Non-Hispanic Black (*n* = 403)	222 (55.1)	181 (44.9)	ref	
Non-Hispanic White (*n* = 157)	77 (49.0)	80 (51.0)	0.81 (0.55, 1.20)	
Hispanic (*n* = 143)	86 (60.1)	57 (39.9)	1.27 (0.88, 1.84)	
Employment				**< 0.01**
Employed (*n* = 188)	128 (68.1)	60 (31.9)	ref	
Unemployed (*n* = 529)	266 (50.3)	263 (49.7)	**2.11 (1.49, 3.00)**	
Anxiety (GAD-7)				**< 0.01**
No (< 10) (*n* = 493)	315 (63.9)	178 (36.1)	ref	
Yes (≥10) (*n* = 219)	79 (36.1)	140 (63.9)	**3.14 (2.25, 4.37)**	
Depression (PHQ-8)				**< 0.01**
No (< 10) (*n* = 492)	308 (62.6)	184 (37.4)	ref	
Yes (≥10) (*n* = 229)	93 (40.6)	136 (59.4)	**2.45 (1.78, 3.37)**	
Post-Traumatic Stress Disorder (PC-PTSD)				**< 0.01**
No (*n* = 511)	315 (61.6)	196 (38.4)	ref	
Yes (*n* = 202)	76 (37.6)	126 (62.4)	**2.66 (1.90, 3.73)**	
Marijuana Use				**0.03**
No (*n* = 466)	266 (57.1)	200 (42.9)	ref	
Recreational use (*n* = 147)	83 (56.5)	64 (43.5)	1.03 (0.71, 1.49)	
Therapeutic use (*n* = 104)	45 (43.3)	59 (56.7)	**1.74 (1.14, 2.68)**	
Alcohol Consumption				0.47
No Drinking (*n* = 192)	112 (58.3)	80 (41.7)	ref	
Non-Hazardous Drinking (*n* = 257)	140 (54.5)	117 (45.5)	1.17 (0.80, 1.71)	
Hazardous Drinking (*n* = 255)	134 (52.6)	121 (47.5)	1.26 (0.87, 1.85)	
Lifetime Injection drug use				0.09
No (*n* = 611)	346 (56.6)	265 (43.4)	ref	
Yes (*n* = 101)	48 (47.5)	53 (52.5)	1.44 (0.95, 2.20)	
Lifetime Crack/Cocaine				0.86
No (*n* = 625)	340 (54.7)	281 (42.3)	Ref	
Yes (*n* = 102)	54 (53.5)	47 (46.5)	1.05 (0.69, 1.61)	

### Mental health factors and any current pain

Differences in mental health symptoms between participants without any current pain and any pain are summarized in Table 1. Among PLHIV reporting any current pain, 63.9% experienced current symptoms of anxiety as compared to 36.1% who did not report current anxiety (*p* <  0.01). Also, among PLHIV experiencing any current pain, 59.4% reported current symptoms of depression compared to 37.4% that did not (*p* <  0.01). Likewise, 62.4% of PLHIV with current pain reported symptoms of PTSD while 38.4% did not report symptoms of PTSD (*p* <  0.01).

### Mental health factors and severe pain

Differences in mental health factors associated with severe pain are summarized in Table 2. Among PLHIV reporting severe pain, 27.4% experienced current symptoms of anxiety as compared to 10.5% that did not (*p* <  0.01). Among participants reporting severe pain, 26.6%, reported current symptoms of depression compared to 10.8% of that did not (*p* <  0.01). Similarly, 27.2% of participants with severe pain reported symptoms of PTSD compared to 11.5% that did not (*p* <  0.01).

**Table 2:** Demographic Mental Health and Substance Use Characteristics of Severe Pain Among PLHIV *N* = 733

Characteristics	Non-Severe Pain *N* = 615 (83.9%)	Severe Pain *N* = 118 (16.1%)	Unadjusted Odds Ratio	*P* Value
Age in Years				**0.03**
18–49 (*n* = 426)	368 (86.4)	58 (13.6)	ref	
≥ 50 (*n* = 307)	247 (80.6)	60 (19.5)	**1.54 (1.04, 2.29)**	
Sex				**< 0.01**
Male (*n* = 500)	435 (87.0)	65 (13.0)	ref	
Female (*n* = 233)	180 (77.3)	53 (22.6)	**1.97 (1.32, 2.95)**	
Race/ethnicity				**< 0.01**
Non-Hispanic Black (*n* = 403)	324 (80.4)	79 (19.6)	ref	
Non-Hispanic White (*n* = 157)	139 (88.5)	18 (11.5)	**0.53 (0.31, 0.92)**	
Hispanic (*n* = 143)	128 (21.7)	15 (13.4)	**0.48 (0.27, 0.87)**	
Employment				**< 0.01**
Employed (*n* = 188)	174 (92.6)	14 (7.4)	ref	
Unemployed (*n* = 529)	427 (80.7)	102 (19.3)	**2.97 (1.65, 5.33)**	
Anxiety (GAD-7)				**< 0.01**
No (< 10) (*n* = 493)	441 (89.5)	52 (10.5)	ref	
Yes (≥10) (*n* = 219)	159 (72.6)	60 (27.4)	**3.20 (2.12, 4.83)**	
Depression (PHQ-8)				**< 0.01**
No (< 10) (*n* = 492)	439 (89.2)	53 (10.8)	Ref	
Yes (≥10) (*n* = 229)	168 (73.4)	61 (26.6)	**3.01 (2.00, 4.53)**	
Post-Traumatic Stress Disorder (PC-PTSD)				**< 0.01**
No (*n* = 511)	452 (88.5)	59 (11.5)	ref	
Yes (*n* = 202)	147 (72.8)	55 (27.2)	**2.87 (1.90, 4.73)**	
Marijuana Use				**0.10**
No (*n* = 466)	400 (85.8)	66 (14.2)	ref	
Recreational use (*n* = 147)	122 (83.0)	25 (17.0)	1.24 (0.75, 2.05)	
Therapeutic use (*n* = 104)	81 (77.9)	23 (22.1)	**1.72 (1.01, 2.93)**	
Alcohol Consumption				0.31
No Drinking (*n* = 192)	165 (85.9)	27 (14.1)	ref	
Non-Hazardous Drinking (*n* = 257)	219 (85.2)	38 (14.8)	1.06 (0.62, 1.81)	
Hazardous Drinking (*n* = 255)	207 (81.2)	48 (18.8)	1.42 (0.85, 2.37)	
Lifetime Injection drug use				0.62
No (*n* = 611)	514 (84.1)	97 (15.9)	ref	
Yes (*n* = 101)	83 (82.2)	18 (17.8)	1.15 (0.66, 2.00)	
Lifetime Crack/Cocaine				
No (*n* = 625)	524 (83.8)	101 (16.2)	Ref	0.90
Yes (*n* = 101)	85 (83.3)	17 (16.7)	1.04 (0.59, 1.82)	

### Substance use factors and any current pain

Additionally, substance use factors associated with any current pain among PLHIV are summarized in Table 1. Of those PLHIV who reported any current pain, 56.7% used marijuana for therapeutic reasons, 43.5% used marijuana for recreational reasons, and 42.9% did not currently use marijuana (*p* = 0.01). Substances including hazardous drinking, lifetime injection drug use, and lifetime crack/cocaine use were not statistically associated with any current pain at the bivariate level.

### Substance use factors and severe pain

Substance use factors associated with severe pain among PLHIV are summarized in Table 2. Among PLHIV reporting severe pain, 22.1% used marijuana for therapeutic reasons, 17.0% used marijuana for recreational reasons, and 14.2% did not currently use marijuana (*p* = 0.04). Substances including hazardous drinking, lifetime injection drug use, and lifetime crack/cocaine use were not statistically associated with severe pain at the bivariate level.

### Sociodemographic factors and any pain

Those PLHIV reporting pain were more likely to be age 50 years or older (51.8%) compared to PLHIV between 18 and 49 years old (40.1%) (*p* <  0.01). PLHIV reporting being unemployed (49.7%) were also more likely to report having pain compared to employed individuals (31.9%) (*p* <  0.0001). Additionally, female participants (49.4%) and non-Hispanic White PLHIV (51.0%) reported greater proportions of pain than their male and non-Hispanic Black counterparts respectively. However, race/ethnicity was not statistically significant at the bivariate level.

### Sociodemographic factors and severe pain

Those PLHIV reporting severe pain were more likely to be aged 50 years or older (19.5%) compared to PLHIV between ages year old 18–49 (13.6%) (*p* < 0.01). Additionally, 19.6% of non-Hispanic Black participants reported severe pain compared to 13.5% of Hispanic and 11.5% of non-Hispanic White participants (*p* < 0.01). Female participants (22.6%) were more likely to report severe pain compared to male participants (13.0%) (*p* < 0.01). Unemployed PLHIV (19.3%) were also more likely to report having severe pain compared to employed PLHIV (7.4%) (*p* < 0.01).

### Any current pain vs. no pain

Results of the adjusted odds ratios for the association of selected mental health, substance use, and sociodemographic factors with any pain are presented in Table 3. Any current pain was significantly more common in participants ≥50 years of age (AOR = 1.73; CI 95% 1.23, 2.45, *p* < 0.01), females (AOR = 1.47; CI 95% 1.01, 2.12, *p* = 0.04), and unemployed participants (AOR = 1.63; CI 95%1.08, 2.45, *p* = 0.02) than PLHIV without those sociodemographic factors. Additionally, any current pain was significantly more common among PLHIV with symptoms of anxiety (AOR = 2.49; CI 95% 1.48, 4.18, *p* < 0.01) or PTSD (AOR = 1.69; CI 95% 1.11, 2.57, *p* = 0.02) compared to PLHIV without those factors. Factors including race/ethnicity, marijuana use, and depression were not significantly associated with any current pain.

**Table 3:** Multivariate Analysis of Selected Covariates of Any Current Pain vs No Pain Among PLHIV *N* = 733

Predictor	Any Pain vs. No pain			*P* value
	Adjusted OR^1^	95% CI^2^		
Age in Years				
18–49		Referent		
≥ 50	**1.73**	**1.23**	**2.45**	**< 0.01**
Sex				
Male		Referent		
Female	**1.47**	**1.01**	**2.12**	**0.04**
Race/Ethnicity				
Non-Hispanic Black		Referent		
Non-Hispanic White	1.40	0.93	2.11	0.11
Hispanic	0.82	0.52	1.30	0.37
Employment				
Employed		Referent		
Unemployed	**1.63**	**1.08**	**2.45**	**0.02**
Anxiety (GAD-7)				
No (< 10)		Referent		
Yes (≥10)	**2.49**	**1.48**	**4.18**	**< 0.01**
Depression (PHQ-8)				
No		Referent		
Yes	0.95	0.57	1.59	0.86
Post-Traumatic Stress Disorder (PC-PTSD)				
No		Referent		
Yes	**1.69**	**1.11**	**2.57**	**0.01**
Marijuana Use				
No use		Referent		
Recreational use	1.07	0.70	1.65	0.74
Therapeutic use	1.55	0.95	2.53	0.08

^1^*OR* Odds Ratio

^2^*CI* Confidence Interval

### Severe pain vs non-severe pain

Results of the adjusted odds ratio of selected factors associated with severe pain are presented in Table 4. Pain was significantly more common in persons ≥50 years of age (AOR = 1.70; CI 95% 1.07, 2.72, *p* = 0.03), females (AOR = 2.02; CI 95% 1.26, 3.24, *p* < 0.01), and participants reporting current symptoms of anxiety (AOR = 2.03; CI 95% 1.03, 4.01, *p* = 0.04) compared to individuals without those factors. On the contrary, pain was protective among Hispanic participants (AOR = 0.48; CI 95% 0.24, 0.96, *p* = 0.04) compared to non-Hispanic Black participants. Factors including marijuana use, employment, depression, and PTSD were not statistically associated with severe pain.

**Table 4:** Multivariate Analysis of Selected Covariates of Severe Pain Among PLHIV *N* = 733

Predictor	Non-Severe Pain vs Severe Pain			*P* Value
	Adjusted OR^1^	95% CI^2^		
Age in years				
18–49		Referent		
≥ 50	**1.70**	**1.07**	**2.72**	**0.03**
Sex				
Male				
Female	**2.02**	**1.26**	**3.24**	**< 0.01**
Race/Ethnicity				
Non-Hispanic Black		Referent		
Non-Hispanic White	0.58	0.32	1.06	0.08
Hispanic	**0.48**	**0.24**	**0.96**	**0.04**
Employment				
Employed		Referent		
Unemployed	1.87	0.96	3.65	0.07
Anxiety (GAD-7)				
No (< 10)		Referent		
Yes (≥10)	**2.03**	**1.03**	**4.01**	**0.04**
Depression (PHQ-8)				
No		Referent		
Yes	1.29	0.66	2.52	0.46
Post-Traumatic Stress Disorder (PC-PTSD)				
No		Referent		
Yes	1.58	0.93	2.70	0.10
Marijuana Use				
No use		Referent		
Recreational use	1.53	0.86	2.71	0.15
Therapeutic use	1.55	0.84	2.85	0.16

^1^*OR* Odds Ratio

^2^*CI* Confidence Interval

## Discussion

This study sought to examine the prevalence of pain in a statewide sample of PLHIV and identify mental health, substance use, and sociodemographic factors associated with having any pain and having severe pain among PLHIV. Our results indicated that roughly half of our sample reported having any pain while 16.1% reported having severe pain during the previous 24 h. Having any pain was positively associated with being older in age, female, unemployed, having current symptoms of anxiety, and PTSD. Additionally, having severe pain was positively associated with being older, female, and having current symptoms of anxiety. As such, the results of this analysis may serve as the foundation for developing pain interventions among PLHIV.

Treating pain among PLHIV is often challenging, as providers may be reluctant to prescribe opioid analgesics for this population [12]. Consequently, researchers have developed nonpharmaceutical approaches to reduce pain among PLHIV. Brandt and colleagues noted PLHIV may benefit from cognitive behavioral therapies (CBTs) targeting pain, depression, and anxiety [34]. CBTs can provide HIV patients with both effective strategies to help manage physical pain (i.e., relaxation strategies and activity pacing), as well as tools to help improve pain outlook, pain acceptance, and pain catastrophizing (e.g., anticipating the worst outcomes), correlates of the physical, occupational, and social consequences of pain [35]. Thus, care providers may consider implementing CBT as a way to reduce pain and adverse mental health outcomes among PLHIV.

### Mental health

Psychological health factors associated with pain are a key concern, as PLHIV are more likely to be diagnosed with anxiety compared to the general population [36]. Our analysis noted symptoms of anxiety were associated with having any pain and severe pain, while symptoms of PTSD were associated with having any pain. These findings indicate a continued clinical need to address psychological distress among PLHIV as previous research has indicated a bidirectional relationship between pain and psychological distress [37]. Our analyses were not able to determine whether the pain, mental health, and substance abuse were causally influencing each other, or whether they could all be part of a common underlying health issue. If pain is contributing to the feelings of anxiety or to the desire to use drugs or alcohol, then treatment of pain may reduce these other symptoms. Nevertheless, it is also possible there is a common underlying mechanism, such as allostatic load. Allostatic load is defined as the process by which life stressors can accumulate leading to the development of medical conditions and is often higher among individuals with chronic conditions such as HIV [38]. As allostatic load increases, demands of life stressors can increase, thus leading to both an increased risk of developing depression and pain. Moreover, psychotherapeutic methods have been shown to be effective in managing both pain and comorbid depression [39].

### Substance use

After controlling for selected covariates, none of the substance use variables were statistically associated with pain. Initially, we expected marijuana use to be associated with pain as chronic pain is one of the leading reasons for starting medical marijuana [40]. Similarly, we thought alcohol may be associated with pain as a recently published qualitative study, Cook et al. concluded that PLHIV may engage in risky drinking, in order to manage physical pain [41]. A similar study by Merlin et al. also noted marijuana use was not statistically associated with reductions in pain among PLHIV [42]. However, studies investigating the associations between marijuana use and pain have reported reductions in pain severity among the general population [43]. Therefore, PLHIV using marijuana for therapeutic reasons may have effectively reduced pain. As such, further research is needed to better elucidate the relationships between marijuana use for pain among PLHIV.

### Sociodemographics

Our study noted that older individuals were more likely to report having any pain and severe pain compared to their younger counterparts. Generally, as people age, they become more likely to report pain. Thus, older PLHIV may be more likely to report nociceptive pain unrelated to HIV status. Additionally, older PLHIV are more likely to report musculoskeletal pain disorders compared to younger individuals. Musculoskeletal pain disorders including arthritis often present with inflammation which may generally be harder to treat, thus resulting in greater pain severity [44]. Women PLHIV were more likely to report having both any pain and severe pain compared to male PLHIV [45]. Furthermore, women often exhibit lower pain thresholds in experimental studies which may explain the increased pain severity in our analysis [46]. Lastly, PLHIV who reported being unemployed were more likely to report having any pain compared to employed individuals. Unemployed individuals may not be insured suggesting unemployed PLHIV may not receive adequate treatment for their pain [47]. Additionally, PLHIV may be unable to work due to their pain, which could adversely affect quality of life.

### Peak-end phenomenon

In order to better control for recall bias and to control for the peak-end phenomenon, we were especially interested in understanding correlates of severe pain intensity. The peak-end phenomenon acknowledges that participant memory wains over time; thus, asking common questions including, “How has your pain been during the past week?” may not adequately capture participants’ pain score [24]. In order to overcome this limitation, we differentiated worse pain intensity by using optimal cut-points from similar studies [30]. Our analysis noted that older age, being female, and having symptoms of anxiety were associated with severe pain. These results suggest that targeting modifiable risk factors for pain, specifically, anxiety may be a crucial step in reducing pain intensity among PLHIV.

### Limitations

A few study limitations are worth discussing. In the current study, the cross-sectional design limits our ability to determine temporality between pain severity and correlates of mental health and substance use. While various recruitment methods were employed, the generalizability of the results may not be applicable to those with private insurance and PLHIV who do not seek healthcare services. Furthermore, we utilized a single measure of pain reliant upon a numerical scale to assess the magnitude of pain and thus may have failed to capture other aspects of pain such as the impact of pain on functioning, or the classification of pain (e.g., neuropathic, somatic). Additional research could incorporate broader measures of pain such as the Neuropathic Pain Scale [48] or the McGill Pain Questionnaire [49] in order to assess the classification of pain as well to measure pain severity. Finally, longitudinal studies are needed in order to better understand the temporal associations between severe pain and various psychosocial factors experienced by older PLHIV who now are living longer because of ART.

## Conclusions

Our study noted roughly half of our participants repored any current pain in the past 24 h. This study contributes to the growing literature on pain among PLHIV by demonstrating that the overlap of syndemic mental health and substance use is associated with increased pain among PLHIV. Additionally, this study identifies modifiable syndemic health factors associated with pain, which may serve as targets for pain interventions. As this trend continues, HIV care providers and researchers should seek to address mental health and drug use correlates of pain among this population.

## Data Availability

The datasets used and/or analyzed during the current study are available from the corresponding author on reasonable request.

## Abbreviations

ART: Antiretroviral Therapy
HIV: Human Immunodeficiency Virus
PLHIV: People living with HIV/AIDS
IRB: Institutional Review Board
DoH: Florida Department of Health
HIPPA: Health Insurance Portability and Accountability Act
OR: Odds Ratio
CI: 95% Confidence Interval
